# Embodied Neuropsychodynamics of the Relational Self Across Space and Time: An Integrative Narrative Review

**DOI:** 10.3390/brainsci16060627

**Published:** 2026-06-11

**Authors:** Sharon Vaisvaser

**Affiliations:** School of Society and the Arts, Faculty of Humanities and Social Sciences, Ono Academic College, 1 Academic Avenue, Kiryat Ono 5560614, Israel; sharon.vaisvaser@ono.ac.il; Tel.: +972-50-7419912

**Keywords:** embodiment, multidimensional self, predictive processing, peripersonal space, interpersonal synchrony, subjective time, autobiographical memory, neuropsychodynamics, psychotherapy

## Abstract

Extensive explorations in neuroscience, psychology, and psychotherapy increasingly recognized the embodied and relational foundations of selfhood, underscoring the need for an integrated framework spanning development, psychopathology, and therapeutic change. This narrative review synthesizes empirical and theoretical literature across neuroscience, embodiment research, predictive processing, developmental science, phenomenology, and psychodynamic theory, proposing a multidimensional neuropsychodynamic framework of embodied selfhood and its clinical implications. A central contribution is the positioning of Peripersonal Space (PPS) as an embodied action-oriented interface that functions as a primary developmental scaffold for bodily self-consciousness, self-other relations, affect regulation and temporal continuity. PPS is proposed as a dynamic matrix linking embodied predictive self-processes with relational experience, thereby shaping subjective temporality and autobiographical processes. Within this framework, subjective time emerges through bodily rhythms, interpersonal synchronization, and predictive engagement with environmental affordances. These embodied temporal processes gradually extend toward autobiographical continuity and mentalizing capacities, supported by coordinated interactions among large-scale brain networks. Psychodynamic concepts including holding, containment, dimensionality, and symbolic transformation are revisited in dialogue with contemporary embodied and relational neuroscience. Clinically, disturbances of selfhood across psychopathological conditions are discussed in relation to altered PPS organization, disturbances in self-evidencing, and embodied temporal continuity. Psychotherapeutic change is conceptualized as involving gradual reorganization across embodied, affective, and reflective dimensions through co-regulation, interpersonal attunement, and temporally extended relational engagement. Overall, this perspective advances a process-oriented and interdisciplinary framework linking embodiment, temporality, autobiographical integration, and psychotherapy, while highlighting directions for future interdisciplinary research at the interface of neuroscience, embodiment and psychodynamics.

## 1. Introduction

Contemporary neuroscience conceptualizes the self as an emergent, embodied process arising from the continuous coordination of neural, bodily, and environmental dynamics. Selfhood is increasingly understood as a form of ongoing organization that integrates sensorimotor activity, interoceptive signals, affective states, and relational engagement into a coherent experiential field. Within this perspective, reflective capacities such as mentalization are increasingly understood as emerging from embodied and relational processes through which internal states become progressively represented, differentiated, and temporally integrated. Bodily experiences thus constitute the primary medium through which the self is enacted, stabilized, and transformed across time [1,2,3].

Recent multidimensional models of selfhood distinguish minimally embodied selfhood and narrative identity, linking these processes to social self-processing, and agentic self-regulation, while suggesting that psychopathology reflects specific disruptions across self-dimensions rather than a unitary self-system [4,5,6,7,8].

Resonating with embodied neuroscience accounts, psychoanalysis has long foregrounded the body as a central substrate of self-experience, progressively highlighting its role in affect regulation, symbolization, and temporal continuity within interpersonal contexts [9,10]. Nonetheless, integrative models that meaningfully bridge neuroscience, predictive frameworks, and psychodynamic theory remain needed. This paper brings these traditions into dialogue, weaving insights from contemporary neuroscience with psychodynamic conceptualizations of self-organization, development, and disruption, as they unfold through relational, bodily, and movement-based processes.

### 1.1. Aims and Rationale

Despite growing interest in embodiment and multidimensional models of selfhood, current frameworks often address bodily self-consciousness, temporality, autobiographical processes, and relational dynamics separately. Likewise, contemporary neuroscience and psychodynamic perspectives have rarely been integrated within a shared spatiotemporal framework of embodied self-organization. The present review addresses this gap by proposing Peripersonal Space (PPS) as an embodied, interactive, action-oriented interface. The paper establishes a conceptual hierarchy wherein PPS functions primarily as a grounded neurocognitive and neuroaffective system that serves as a developmental scaffold for the coordination of bodily self-consciousness, affect regulation, and self-other relations, ultimately enabling the emergence of temporally extended forms of narrative and reflective self-experience.

Integrating contemporary neuroscience with developmental, phenomenological, and psychodynamic perspectives, the framework explores how the organization of near-body experience may contribute to the development of embodied selfhood, subjective temporality, autobiographical continuity, self-other differentiation, and alterations in embodied self-experience across psychopathological conditions. The review further examines how these embodied, temporal, and relational processes may inform psychotherapeutic reorganization and integration. In doing so, the paper aims to advance a multidimensional neuropsychodynamic account of embodied selfhood across bodily, temporal, and relational dimensions.

### 1.2. Integrative Scope and Literature Selection

The article presents an integrative narrative review aimed at synthesizing contemporary perspectives on embodied selfhood across neuroscience, predictive processing, developmental science, phenomenology, psychodynamics, psychopathology, and psychotherapy.

The literature discussed was selected through iterative, concept-driven searches across major scientific databases, including PubMed, PsycINFO, and Google Scholar, with emphasis on contemporary empirical and theoretical contributions aligned with the review’s conceptual aims. Search terms included combinations of concepts such as “peripersonal space”, “embodied self”, “predictive processing”, “active inference”, “interoception”, “bodily self-consciousness”, “subjective time”, “autobiographical memory”, and “interpersonal synchrony”. Foundational psychoanalytic and phenomenological texts were included based on their conceptual relevance to embodied and relational models of the self, particularly in relation to notions of holding, containment, dimensionality, and symbolic transformation.

Several hundred articles were initially considered across these domains, with selection guided by theoretical relevance, empirical contribution, interdisciplinary significance, and direct relation to the review’s integrative aims. Priority was given to peer-reviewed articles, systematic reviews, theoretical models, and recent interdisciplinary work published primarily within the last 10–15 years, while also incorporating seminal psychoanalytic and phenomenological contributions where theoretically relevant. These works were included as historically influential conceptual frameworks that continue to inform contemporary embodied and relational accounts of selfhood and psychotherapy. Included sources were selected based on their conceptual and empirical relevance to the integrative aims of the review, particularly regarding embodied self-organization, PPS, temporality, relational processes, and psychopathology. Studies that were only tangentially related to these themes or did not substantially contribute to the proposed framework were excluded. In areas where empirical findings remain limited or heterogeneous, particularly regarding psychodynamic and clinical interpretations, the manuscript adopts a heuristic and hypothesis-generating perspective rather than asserting definitive mechanistic conclusions. No formal risk-of-bias assessment, evidence grading, or quantitative synthesis was conducted, consistent with the narrative scope of the review.

Given the conceptual breadth of the fields discussed, the present review does not aim to resolve theoretical differences or provide exhaustive coverage of competing models. Instead, it focuses on identifying points of convergence across contemporary neuroscience, embodiment research, predictive processing, and psychodynamic perspectives that may support an integrative understanding of multidimensional selfhood. The proposed framework is therefore intended as a heuristic and interdisciplinary model designed to stimulate further empirical, theoretical, and clinical inquiry. The article is organized progressively, moving from the theoretical foundations of embodied self-organization to the role of peripersonal space in shaping bodily selfhood, affect regulation, and self-other relations, before extending toward subjective temporality and autobiographical continuity. Perspectives on psychopathology and psychotherapeutic processes are integrated throughout these different levels of analysis.

## 2. Theoretical Foundations of Embodied Self-Organization

Contemporary embodied perspectives increasingly conceptualize selfhood as emerging through ongoing bodily and relational processes. These views situate self-organization within hierarchical inferential dynamics, whereby embodied agents continuously regulate internal states and exchanges with the environment through predictive, action-oriented processes. Within this framework, affective and cognitive functions remain fundamentally grounded in embodied experience and interpersonal engagement, carrying important implications for psychotherapeutic processes.

### 2.1. Predictive Organization of the Embodied Self

Bayesian predictive processing and active inference frameworks provide a principled account of this embodied organization. According to these frameworks, the brain continuously generates predictions about incoming sensory signals and actively (mostly enactively) selects actions that align sensory input with anticipated states [11]. Prediction errors emerging from mismatches between expected and incoming sensory signals drive the ongoing updating of internal models and support adaptive engagement with changing bodily, relational, and environmental conditions. This process involves the precision-weighting of sensory signals, from within the body and the surrounding environment, whereby inputs are differentially amplified or attenuated according to contextual relevance and affective salience. Through recurrent loops of perception and action, the individual as an embodied agent infers the causes of ongoing sensations and maintains adaptive and developing engagement with the environment.

The embodied self can be described as a self-organizing system whose functioning relies on statistical boundaries, commonly framed as *Markov blankets*, that differentiate inside from outside and self from non-self across multiple experiential levels. These spatial, ontological, autopoietic boundaries support the experience of being a distinct yet relational subject and enable the construction of meaning from an ongoing flux of sensory and affective signals [12]. In this sense, the concept of Markov blankets may be considered a heuristic parallel to Freud’s notion of the protective screen, insofar as both describe boundary-forming processes that regulate exchanges between internal states and the external environment. These frameworks, however, emerge from distinct epistemological traditions and are presented here as heuristic correspondences [9].

Within this framework, affective intentionality refers to the way bodily and affective states organize perception, salience, and action tendencies in relation to the environment and to others. Through these embodied processes, affect contributes to the regulation of self-environment boundaries and guides adaptive engagement with the world. In doing so, affective intentionality orients the organism toward or away from preferred states of living, shapes what is experienced as threatening or supportive, and guides action within a shared relational space [13]. From this perspective, the self may be understood as an emergent and dynamically regulated process that maintains relative coherence through embodied action and relational engagement. Within predictive frameworks, this ongoing confirmation of embodied existence has been conceptualized as self-evidencing [14]. From an experiential perspective, phenomenal self-modeling has been proposed to contribute to minimal forms of self-consciousness, reflected in the pre-reflective sense of being an agent situated within and actively shaping its sensorium while maintaining an embodied presence in the world through self-evidencing action [15,16].

Crucially, predictive inferences are grounded in embodied organization. Recurrent sensorimotor patterns, movement capacities, visceral rhythms, and affective signals shape how predictions are formed and constrain the range of actions available to the embodied agent [17]. Bodily self-consciousness is based on the integration of multisensory inputs and motor signals, and includes the sense of agency, ownership, first-person perspective, and self-location [18]. Early in development, repetitive cycles of movement, touch, and sensory feedback establish foundational models of bodily continuity and agency. These early embodied regularities support the gradual formation of more differentiated self-models that integrate perception, action, and anticipation. Variations or disruptions in these embodied and relational processes may contribute to altered forms of bodily self-consciousness, affect regulation, and interpersonal organization.

### 2.2. Affective and Cognitive Dynamics in Embodied Relational Self-Organization

Contemporary embodied accounts increasingly converge in conceptualizing affect, interoception, and action as central organizing processes of selfhood. At the intersection of predictive processing and neuropsychoanalytic accounts, drives may be understood as biologically grounded demands that generate deviations from expected bodily states. Affects, in turn, constitute the felt, value-laden experience of these deviations, translating prediction error into motivational signals that guide action, regulate self-environment boundaries, and support ongoing self-organization [19]. Recent neuropsychoanalytic formulations further clarify how unconscious affective drives shape hierarchical predictive models by modulating precision-weighting, thereby biasing interoceptive inference toward motivationally salient states [20].

From a complementary perspective, emotions may be conceptualized as constructed interoceptive predictions shaped by prior experience, contextual inference, and ongoing bodily regulation [21,22]. Prediction errors generated through mismatches between predicted and actual bodily states contribute to emotional allostasis and ongoing self-awareness [23]. These mechanisms extend beyond internal predictive error minimization to embodied engagement with the environment, aligning with 4E (embodied, enacted, embedded, and extended) approaches to cognition that conceptualize perception, thought, memory, and imagination as grounded in embodied and relational interaction with the world. Cognition reflects hierarchically organized inferential processes through which embodied agents continuously update generative models of self and world. Accordingly, mental processes such as perception, attention, reflection, memory, and imagination constitute dynamic forms of model updating and precision-weighting, grounded in bodily regulation and affective orientation. Even higher-order forms of thought thus emerge from and remain anchored in embodied and relational engagement, enabling experience to acquire coherence across contexts and over time.

Together, these frameworks highlight the centrality of bodily dimensions in psychotherapeutic processes [24]. Embodied patient–therapist interactions may complement verbal interpretation through sensory, motor, affective, and relational processes that support co-regulation and experiential reorganization. Patient–therapist enactments critically extend interpretation, whereby embodied “witnessing” and relational processes co-create potentially transformative subjective experiences, a transformative self-organization beyond verbal understanding [25]. The therapeutic relationship may involve dynamic movements between experiential connectedness, including moments of experiential unity described as “oneness” [25] or “at-one-ment” [26], and processes of self-other differentiation, that support both relational safety and psychological autonomy. Viewing the body as a multidimensional system thus provides a foundation for integrative and personalized psychotherapeutic interventions.

The following sections further develop these embodied and relational perspectives through an examination of PPS and its proposed role in the organization of bodily selfhood, affect regulation, subjective temporality, autobiographical continuity, and psychotherapeutic transformation.

## 3. The Multidimensional Body: Peripersonal Space as a Matrix of Self-Representations

A key domain in the neuroscience of selfhood concerns the Peripersonal Space (PPS), the near-body spatial zone immediately surrounding the body. Closely related to the notion of the Kinesphere, PPS constitutes a lived, experiential field in which sensory signals and action possibilities are tightly coupled, enabling rapid inference, regulation, and action readiness [27]. Functioning as a multisensory interface between the body and its immediate environment, PPS contributes to the organization of bodily boundaries, motor intentions, and relational orientation, while serving as a primary medium for emotional expression and the felt sense of presence [2,27]. In this way, PPS may provide an embodied scaffold for aspects of situated conscious experience, shaping what is experienced as immediately present, self-relevant, and available for action. Accordingly, PPS representations have been linked with components of bodily self-consciousness [28]. PPS is thought to dynamically reorganize according to social proximity, trust, threat, and the affective meaning of others, thereby contributing fundamentally to how the self is situated in relation to the world and to others, guiding approach and avoidance behaviors [29,30].

To clarify the conceptual architecture of the proposed framework and distinguish it from existing multidimensional models of the self, the review establishes a clear hierarchy among the various roles of PPS. Primarily, PPS is conceptualized here as a specific and dynamic sensorimotor–affective interface. Crucially, it is proposed that this neurocognitive and neuroaffective action-oriented system serves as a developmental scaffold for higher-order self-processing. The framework further positions PPS as the foundational matrix upon which more complex, multidimensional layers of selfhood, such as the reflective (mental) and narrative self, are progressively constructed. Consequently, PPS transitions from a low-level sensorimotor boundary into a broader phenomenological field of relational experience, providing a heuristic bridge to psychodynamic concepts and serving as a potential transdiagnostic marker in psychopathology when its plastic boundaries become rigidified or blurred.

### 3.1. Neurodynamics of the Peripersonal Space

At the neural level, PPS emerges from the activation and connectivity of distributed regions, including the associative premotor cortex, the sensorimotor and somatosensory networks, and the dorsal attention network. The involvement of the salience network, anchored in the anterior insula and anterior cingulate cortex, enables multisensory integration and attribution of perceptual salience and attention to internal or external emotionally relevant (including social) stimuli. Subcortical regions such as the putamen, basal ganglia, and the cerebellum contribute to motor calibration, action selection, and timing processes that refine anticipatory responses within near-body space. Relay stations like the pulvinar thalamus and superior colliculus integrate tactile, visual and auditory inputs through recurrent sensorimotor loops that support attentional orienting, motor preparation, and the selection of contextually appropriate actions within PPS [31]. Importantly, dynamic patterns of functional connectivity among these regions enable flexible updating of bodily boundaries and action relevance in accordance with changing interoceptive states and environmental demands, while mediating the interaction between PPS representations and higher-order affective and cognitive functions.

Therefore, PPS is experienced as directly available for action and is shaped by internal states as well as contextual factors carrying emotional, social, gendered, and cultural meaning [29,32]. It supports communicative movement, goal-directed action, and protective responses, capacities frequently altered in disorders of the self. Conceptualized as an action-oriented field, PPS encodes the relevance and value of potential actions, enabling anticipation and prediction through the continuous updating of internal models of self–environment interaction [33,34].

### 3.2. Developmental Emergence of PPS

Developmentally, the foundations of this bodily-experiential field are laid early. During fetal life, rhythmic exteroceptive inputs, such as maternal heartbeat, breathing, blood flow, and voice, are integrated with spontaneous self-generated movements and tactile contact with the uterine environment [35]. Maternal movements provide additional somatosensory and vestibular stimulation that contributes to cortical sensorimotor development and early embodied representations of the body in space [36]. These processes involve implicit inter-rhythmic synchrony between maternal and fetal physiological patterns, supporting the emergence of bodily self-awareness. Recurrent loops of action and sensory feedback, grounded in tactile and proprioceptive experience, are facilitated by developing connectivity among brainstem arousal systems, limbic affective circuits, and thalamo-cortical pathways, giving rise to proto-representations of the body in space and early sensory learning that shapes postnatal experience [37].

The infant bodily self is co-created through embodied relational processes, in which moments of rupture and repair in early interaction play a primary role in shaping bodily self-experience, with reparation initially achieved through tactile contact [38]. Affectionate caregiver skin-to-skin touch, mediated by C-tactile afferents, further shapes PPS maturation by promoting parent–infant neural synchrony and oxytocin release. These effects are supported particularly by the anterior insula, one of the earliest cortical regions to develop and differentiate, which integrates interoceptive signals and plays a key role in transforming immediate bodily sensations and tactile self-other proximity into more sustained representations mediated by gaze and imagery [39]. Indeed, as multisensory integration matures during development, bodily self-experience becomes increasingly differentiated, transitioning from surface-based tactile–proprioceptive organization toward integrated visuospatial representations [28,40].

Psychodynamic accounts offer a complementary view of this process, conceptualizing it as a progression from a predominantly *tactile envelope* of the self toward increasingly visual and symbolically representational modes of self-experience [41,42]. Bodily holding and touch gradually support the capacity to maintain representations of self and other in the absence of direct physical contact, mediated through gaze, imagery, and anticipated movement [43].

From this early developmental stage, mirror-related neural systems play a contributory role in organizing PPS as an intrinsically relational field. Mirror mechanisms, encompassing premotor, parietal, and somatosensory regions, as well as salience network regions, such as the insula, are activated both during the execution of actions and the observation of others’ movements. These systems enable action–perception coupling and embodied resonance and simulation, supporting the infant’s embodied sensitivity to others’ movements, intentions, and affective expressions as behaviorally and relationally relevant to the self [44,45]. In this sense, mirror-related processes provide a pre-reflective scaffold for self-other differentiation, supporting early forms of intersubjective attunement that precede symbolic representation and explicit mentalization.

Supported by mirror mechanisms, recent evidence positions PPS as an inherently relational, co-constructed field that is continuously reorganized through embodied interaction. Within this field, transient expansions and partial overlaps facilitate interpersonal bonding, while subsequent re-differentiation upholds bodily boundaries and self-other distinction [46]. This dynamic may also resonate with psychotherapeutic processes involving oscillations between experiential connectedness and differentiated self-other relating. The following sections extend the framework into clinical domains, combining empirical findings with theoretically informed interpretations and hypothesis-generating extensions. Importantly, the framework integrates empirical findings and psychodynamic interpretations across multiple levels of embodied self-processing. Moreover, the specificity and strength of current evidence vary across conditions, with some claims more directly supported by PPS research and others relying more broadly on findings related to bodily self-consciousness, interoception, affective regulation, and relational self-experience.

### 3.3. PPS in Psychic Organization and Psychopathology

From a psychodynamic and phenomenological perspective, PPS may provide an embodied spatial framework through which different modes of self-organization and self-other relatedness can be explored. In early organization, experience has been postulated to be dominated by immediacy, surface, contact, and proximity, relying primarily on sensorimotor impressions, akin to psychodynamic notions of adhesive identification, where self-cohesion depends on “sticking” to an external surface (in the environment or the caregiver/therapist) without internalized containment or depth [47].

As integration deepens, PPS may support greater differentiation, depth, and flexibility in self-other relations. Thus, PPS contributes to embodied forms of self-organization ranging from predominantly surface-bound to more differentiated and flexible modes of relational experience [48]. Clinically, pathological rigidity in the predominantly surface-bound PPS organization may resemble psychodynamic descriptions of “second skin formation”, a defensive muscular/hypervigilant envelope substituting for failed psychic containment, restricting PPS flexibility and relational depth [41,47].

From an embodied predictive perspective, such surface-bound and sensory-dominated modes of organization can be understood as constrained forms of self-evidencing, in which the self confirms its existence through immediate bodily sensation, repetitive action, or heightened salience [49,50]. Disruptions of the minimal pre-reflective self, described across a range of psychiatric conditions, including psychosis, autism spectrum disorder, personality disorders, and trauma-related states, are characterized by fragility of first-person perspective, altered sense of agency or ownership, and instability of self-other boundaries. In these contexts, altered integration between interoceptive and exteroceptive signals may be associated with a tendency to rely more heavily on predictable sensorimotor engagement, such as motor repetition or sensory self-stimulation, as compensatory strategies that may help stabilize fragile self-coherence by minimizing uncertainty and sustaining a sense of presence through bodily immediacy.

In typical development and mental health, the boundaries of PPS and the transitions between peripersonal and extrapersonal space (the environment or the other) gradually become characterized by flexibility and context sensitivity. By contrast, disturbances in the organization of this space have been documented across various self-related conditions. In the autism spectrum, studies suggest alterations in PPS boundaries and multisensory integration, often characterized by reduced PPS flexibility and sharper boundaries, reflecting a rigid differentiation between self and other [48]. Meltzer associated such autistic states with a two-dimensional mode of experience characterized by surface-bound forms of relational engagement and limited experiential depth.

In the schizophrenia spectrum, findings have been linked to diffuse or blurred PPS boundaries in social context, indicating a weakened self-other distinction [48,51]. These altered bodily and spatial experiences may partially resonate with Meltzer’s description of pseudo three-dimensionality and claustral dynamics, in which experiences of connection and separation appear to fluctuate between closeness and distance, while both genuine relatedness and true separateness remain impaired. Meltzer conceptualized the claustrum as a phantasized internal psychic space in which the self becomes experientially enclosed within the object, accompanied by distorted bodily experience and unstable self-other boundaries [52]. Although Meltzer’s formulations emerged from psychoanalytic clinical observation, contemporary research on PPS and embodied self-organization may help illuminate aspects of the experiential phenomena he described.

In post-traumatic stress disorder, an expanded PPS has been associated with heightened threat vigilance, while dissociative states are characterized by instability, displacement, and loosening of spatial boundaries [53]. Meltzer noted the “breakdown of surfaces” caused by relational trauma. Recent evidence suggests that social motor cooperation can flexibly reshape PPS boundaries in ways modulated by trait anxiety, pointing to the therapeutic potential of embodied interpersonal attunement in anxiety-related disturbances of self-boundary [54].

Although empirical evidence increasingly implicates alterations in PPS organization across several psychopathological conditions, the strength and specificity of current findings vary considerably between disorders. Evidence is strongest in schizophrenia spectrum conditions, anxiety and trauma-related disorders, and autism spectrum conditions, whereas broader transdiagnostic and psychodynamic implications remain more exploratory and hypothesis-generating.

Sex and gender identity further exemplify the plasticity and social modulation of embodied selfhood within PPS. PPS boundaries have been proposed to interact with gendered embodiment and culturally shaped self-experience, potentially influencing body ownership, agency, and self-other relations [7]. Recent neurocognitive models have begun to frame gender fluidity and non-binary identities as emergent properties of neural complexity and predictive plasticity, aligning with depathologizing frameworks [55]. From this perspective, tensions between embodied self-experience, bodily self-representation, and socially mediated gender experience may contribute to forms of dysphoria and embodied distress, underscoring the potential role of psychotherapy in supporting experiences of embodied self-continuity through relational attunement. However, empirical evidence directly linking PPS organization to gender dysphoria remains preliminary and requires further investigation.

Taken together, these observations suggest that PPS may contribute to the embodied organization of self-models and self-other relations in the present moment and may provide a dynamic scaffold through which the subjective experience of continuity, anticipation, and change unfold across time.

## 4. From Peripersonal Action Space to Embodied Subjective Time

Peripersonal near-body space has been conceptualized as an action-oriented field that encodes the value and relevance of potential actions [32]. This organization is inherently temporal, structured around anticipation, readiness, and the continuous projection of action into the near future, supporting prediction through updated self-environment models. Within the present framework, temporal organization is approached as operating across multiple interacting scales, ranging from immediate sensorimotor anticipation within PPS, through embodied affective and interpersonal rhythms, to episodic autobiographical memory and temporally extended narrative self-processes.

### 4.1. Temporal Dynamics of PPS

The PPS is represented in the brain as a dynamic, egocentric value map, in which objects, directions, and distances are encoded relative to the body according to their relevance for action and internal needs [56]. Because these representations are continuously revised across unfolding sensorimotor cycles, they may support immediate forms of embodied temporal continuity.

Importantly, such dimensional expansion is both intrapsychic and interpsychic. Advances in second-person neuroscience emphasize that temporally extended self-experience is co-constructed within interpersonal interaction. Through embodied engagement, affective attunement, and interpersonal synchronization, predictive models are aligned within a shared relational field, contributing to the joint emergence of meaning and self-experience [57,58]. This inter-brain synchronization has been associated with a subjective sense of social connectedness, engagement, and cooperativeness, as well as experiences of cohesion and self-other merging. The constitution of this participatory, extended form of consciousness challenges individualistic models by demonstrating collective binding of subjective experience through shared oscillatory dynamics [59].

Many forms of psychopathology are associated with a reduced capacity for brain-to-brain synchrony, often accompanied by diminished behavioral and interpersonal coordination during social interaction [53,54]. Disruptions in interpersonal neural alignment and attunement have been linked to altered relational processing across mental disorders, reflecting impaired dyadic regulation. Converging systematic reviews indicate that psychotherapy has been associated with neural, physiological, and behavioral alignment between patient and therapist, and that repeated relational synchrony may promote experience-dependent plasticity, thereby strengthening interpersonal synchronization capacities [55,56,57]. Clinically, these findings may help illuminate how embodied dyadic co-regulation contributes to therapeutic change within a shared relational field.

Interpersonal neural synchronization in dyadic rhythmic interaction, peaking in the salience and reward networks through the involvement of oxytocin and dopamine, respectively, temporalizes PPS predictions, linking behavioral rhythms to shared brain states scaffolding self-continuity [60]. These value-weighted representations are continuously updated by contextual, emotional, and interoceptive states, shaping perception, attention, and action readiness prior to explicit conscious processing. A sense of dynamic continuity across successive moments of perception and action enables adaptation to novel experiences and supports rapid, efficient interaction with a constantly changing environment.

Subjective time emerges as an embodied process through the regulation of action across unfolding cycles of perception and movement, grounded in bodily rhythms (e.g., cardiac and visceral signals) and ongoing sensorimotor flow. These embodied temporal processes engage key neural hubs, mainly the bilateral insula, cerebellum, and supplementary motor area, where interoceptive and sensorimotor signals converge to represent duration [61,62]. Disruptions in these processes affect perception, emotion, and cognition across a range of clinical conditions, emphasizing the centrality of embodied temporal continuity for self-organization [63]. Within this framework, consciousness may be conceptualized as the subjective organization of embodied temporal processes that sustain continuity of self-experience across time [64].

### 4.2. Embodied Mechanisms of Subjective Time

Self-consciousness depends on the ongoing generation of predictive models that enable the individual to anticipate the consequences of action and to project themselves forward in time. Conscious experience is therefore “temporally thick”, integrating bodily traces of the past, engagement in the present, and anticipatory orientations toward the future within ongoing embodied action [65]. Through such prospective inference, actions are selected so as to reduce future uncertainty, supporting a coherent sense of agency and continuity. This thickness of temporal organization of experience captures the embodied capacity to integrate embodied traces of the prior experience, engagement in the present, and future-oriented anticipation within ongoing experience. Self-evidencing thus includes counterfactual processing, i.e., what might have happened or could yet occur, often simulated through bodily sensations and movement imagery [65]. Phenomenologically, this aligns with accounts of time as a continuous flow, in which retention (directed towards an immediate past), the present moment, and protention (directed towards the immediate future) are dynamically intertwined in the “specious present” [66].

Temporally extended self-experience is grounded in the perception of environmental affordances, organized within the PPS, that is, the possibilities for action that the environment makes available to the embodied self [66]. Through ongoing engagement with these affordances, experience acquires continuity across time, resonating with Winnicott’s notion of *going on being*, which emphasizes a sustained sense of selfhood supported by environmental holding and relational stability [67]. In this view, continuity of experience depends on the ongoing availability of action possibilities that allow the self to remain open, responsive, and temporally extended.

Within active inference accounts, this openness is formalized through the selection of policies or courses of action that minimize expected free energy, corresponding to a reduction in anticipated surprise and uncertainty about outcomes [11,68].

### 4.3. Rigidity, Repetition, and Therapeutic Rhythm

When egocentric value maps of the near-body environment become rigid or over-weighted by threat, the range of perceived affordances narrows, biasing action selection toward repetitive, low-uncertainty policies, even when such policies are maladaptive [54,69,70]. At the level of experience, this restriction is accompanied by alterations in consciousness, including temporal freezing, narrowed awareness, and a diminished openness to alternative possibilities for action and meaning.

Considering this, Freud’s notion of repetition compulsion, the paradoxical tendency to re-enact familiar distressing patterns [71], may be understood as an attempt to preserve predictability within the peripersonal action field by constraining exploration and anchoring the agent to familiar sensorimotor trajectories. Psychoanalytic accounts further elaborate repetitive patterns of experience in terms of disrupted symbolic transformation and temporal integration. Bion described repetition as the persistence of unprocessed emotional and sensory (beta) elements that remain outside symbolic thought and temporal sequencing, left without containing (alpha) function [72]. Winnicott emphasized how failures in early holding and continuity of being may lead experiences to recur as enacted states rather than remembered events [73].

Accordingly, relational developmental accounts offer a complementary articulation of the neurobiological processes of subjective time. Colarusso described the emergence of time sense as a gradual developmental achievement, grounded in bodily rhythms, affective regulation, and the growing capacity to tolerate absence as object constancy is established [74]. Similarly, Arlow emphasized that psychic time is an experiential dimension shaped by continuity, anticipation, and meaning, closely tied to emotional development and the organization of internal object relations [75]. From this perspective, the experience of self-continuity in time, through ongoing self-evidencing processes, resonates with the developing capability of object constancy.

The centrality of subjective time experience, grounded in ongoing body movement within intersectional social contexts, underscores the importance of attending to temporal dynamics in clinical contexts. The therapeutic environment must remain sensitive to the rhythm and unfolding of events within the therapy room, to the sense of continuity or interruption, and to patterns that appear frozen in time or repetitive and circular in nature [76]. Attention to bodily rhythm, movement flow, and moments of synchrony, or rupture and repair, may strengthen the integration of experience over time, supporting the development of more coherent and flexible self-models.

## 5. Spatiotemporal Expansion: Autobiographical Memory, Mental Self, and Four-Dimensional Relational Experience

Beyond immediate and rhythmically structured forms of temporal experience, selfhood further develops reflective and autobiographical modes of temporal organization. Building on embodied temporal continuity, self-experience further develops into a reflective and representational domain commonly described as the autobiographical [77], narrative [78], or mental self [79]. At this level, the self acquires the capacity for *mental time travel*, i.e., the ability to flexibly shift perspective across past, present, and future by reactivating and recombining autobiographical representations. This capacity supports remembering past experiences, simulating possible futures, and organizing lived experience into coherent temporal sequences [80].

### 5.1. Narrative Extensions of Embodied Time

At this extended autobiographical level, through temporally mobile mode of consciousness, experience becomes explicitly reflective and narratively structured, allowing the self to be experienced as extended and continuous, while remaining grounded in ongoing embodied presence. Within this framework, autobiographical memory constitutes an active mechanism through which individuals organize, interpret, and integrate their lived experiences into a coherent and meaningful sense of identity. Episodic memory was suggested to emerge from predictive processing mechanisms, whereby the brain actively constructs temporal experience through the integration of past sensory predictions, present perceptual updating, and future-oriented inference [81]. Recent findings further demonstrate premotor–hippocampal coupling during episodic memory retrieval, suggesting that motor-related representations of the bodily self-present at encoding are neurally reinstated when past episodes are recalled [82]. This coupling highlights how autobiographical memory remains grounded in action-oriented bodily states, enabling a coherent sense of self extended across time.

The mental dimension of the self is supported by the Default Mode Network (DMN), encompassing the medial prefrontal cortex (mPFC), posterior cingulate cortex (PCC), precuneus, inferior parietal lobule and the hippocampal formation, which dynamically integrates prior experience, bodily states, and social information into context-rich predictive models of unfolding events across time [83,84]. Accordingly, DMN has been implicated in emotional processing, mind-wandering and spontaneous thought, episodic future thinking, and mentalizing. Recent studies frame the DMN as a generative engine for self-relevance and self-reference, continuously simulating embodied predictions of personal salience within sensorimotor and interoceptive contexts to construct coherent narrative continuity. These mental operations remain anchored in embodied repertoires, with bodily interoceptive and autonomic fluctuations actively modulating DMN-mediated reflective processes through functional connectivity with salience and reward networks, e.g., the anterior insula and ventral striatum, respectively [85]. The anterior insula detects salient bodily and social stimuli, enabling flexible transitions between DMN-mediated internal reflection, including autobiographical memory retrieval and coherent narrative self-construction, and externally oriented focus via the central executive network [86]. This is a mechanism that is disrupted in psychiatric disorders [87].

### 5.2. Embodied Ground of Mentalization

Investigations of the reflective function have identified overlapping neural circuits underlying mentalizing processes concerning one’s own internal bodily and mental states, and those of others, particularly in hubs of the DMN and the temporoparietal junction. Accordingly, both the sense of self and the sense of others rely on the functional integration and segregation of default mode, sensorimotor, salience and executive brain networks [5]. These findings may further suggest that the capacity to reflect on one’s own internal experience provides an important developmental and neural scaffold, for resonating and understanding others’ states [88]. Moreover, the capacity to mentalize emerges through being seen and mentalized by another. Being wired for social connectedness, the self comes to feel and know itself through the other’s mirroring and reflective stance. This relational matrix progressively scaffolds the mental self’s temporal agency, enabling fluid mental movement through autobiographical timelines while preserving embodied continuity across intersubjective encounters.

From this perspective, recent accounts of active intersubjective inference propose that the self emerges through recursive, second-order predictions about how one is perceived by others, dynamically interacting with interoceptive priors to regulate bodily–social boundaries and offering a predictive framework for psychodynamic phenomena such as transference and projection [89].

Developmental neuroscience substantiates the link between the neural underpinnings of the self and the social world, highlighting the central role of attachment relationships in shaping the neural architecture underlying the nested, multidimensional self. These developmental findings provide a crucial bridge to understanding how the self expands across time toward reflective and mnemonic dimensions. Work on social brain development highlights the protracted maturation of large-scale networks, such as the default mode and salience networks, and their sensitivity to early caregiving experience [90]. Longitudinal evidence further indicates that early synchronous caregiving tunes the social brain in ways that persist across the lifespan, recruiting distributed networks involved in self-referential processing, affect regulation, and memory integration from infancy through adulthood [91,92]. This pattern is consistent with the internalization of co-regulation and shared rhythms as embodied expectations that support reflection and temporally extended self-experience. These interactions scaffold internally generated reflection, autobiographical organization, and flexible temporal perspective-taking through repeated embodied interactions with attachment figures. Through affective attunement, co-regulation, and shared rhythms, caregiving relationships are thought to shape developing neural organization in ways that support the emergence of an embodied, temporally extended, relationally grounded mental self [93].

The relaxed state that enables mentalizing, playful daydreaming or mind-wandering is crucial for psychotherapy. The phenomenological qualities associated with DMN-mediated state of mind may bear resemblance to Winnicott’s concept of non-integration in transitional space, a developmental achievement that presupposes a safe sense of integration, and allows an integral sense of self to arise [94,95]. Through accumulating embodied interactions, the child internalizes environment–mother experiences and patterns of caregiver presence, as sensorimotor, affective, and interoceptive regularities. These are gradually carried over into expectations, or predictions, regarding safety and availability, shaping autonomic tone and action readiness. Such embodied safety affords the freedom to let the mind wander, allowing reflective and autobiographical processes to unfold while remaining anchored in bodily and interoceptive signals. In this sense, Winnicott’s notion of the *capacity to be alone* offers a complementary psychodynamic lens on the developmental achievement of a temporally extended embodied self-experience: the ability to remain with one’s internal bodily-affective experience in the absence of immediate external regulation. This capacity, based on the trust and continuity of the symbolic presence of the internalized object, allows the self to sustain absence and engage in reflective and imaginative activity and thought [95,96].

### 5.3. Dimensionality, Disruption, and Therapeutic Integration

Meltzer’s theory of dimensionality offers yet another psychoanalytic articulation of this qualitative shift in temporal self-experience [97]. As self-related processing becomes increasingly mediated by mental time travel, the self elaborates the capacity to tolerate absence, anticipation, and symbolic transformation across time. While earlier modes of organization (one- and two-dimensionality) emphasize rhythmic immediacy, surface contact and planar spatial differentiation without depth, three-dimensional experience introduces containment of internal objects. This, in turn, paves the way for a four-dimensional experience, which entails the capacity to inhabit psychic life as extended in depth, space, and time. This mode allows the self to be sustained across past, present, and future while remaining open to revision and change.

Contemporary neuroscience offers points of convergence with this formulation, as exemplified by the recent Self-Simulational Theory of temporal extension [98]. According to this account, four-dimensional self-experience emerges from the dynamic tension between the *self-as-actual*, anchored in the embodied present, and the *self-as-counterfactual*, which enables mental time travel through prospective simulation. This capacity is mediated by large-scale brain connectivity linking self-referential and interoceptive processes, thereby framing four-dimensionality as the ability to sustain temporally extended and context-sensitive self-models grounded in episodic autobiographical memory.

From a Bionian perspective, the emergence of temporal self-experience that encloses a symbolic capacity depends on the capacity to transform lived experience into memory and thought, allowing past states to be held, revisited, and modified in light of present meaning [72]. A heuristic parallel may be drawn between Bion’s notion of alpha function and contemporary accounts of hippocampal–DMN integration involved in transforming immediate affective experience into temporally organized autobiographical representations. Within this perspective, raw beta-elements may be understood as progressively transformed into symbolic and thinkable experience, resonating with neurocognitive accounts describing how episodic memories gradually become integrated into coherent autobiographical narratives through coordinated interactions between the hippocampus and DMN hubs, including the mPFC and PCC [99,100].

Traumatic experiences disrupt this cohesive memory process, altering the registration of these events to memory across body, time, and context. Under conditions of amygdala-driven overwhelming arousal, lived experience is encoded as salient bodily states. These embodied traces carry highly weighted threat-related predictions that orient perception, affect, and action toward heightened bodily readiness and defensive coordination [101,102,103]. These predictive configurations privilege immediacy, action potential, and interoceptive salience, thereby structuring self-experience around embodied relevance. The fragmented re-experiencing of trauma has been linked with altered activation and diminished connectivity within and between the DMN and other regions [104]. Relational and attachment-based trauma is associated with increased recruitment of salience and sensorimotor networks, reflecting a mode of self-experience grounded in affective intensity, with reduced reliance on higher-order symbolic mediation [105].

Overactivation of the DMN, together with imbalanced communication with the executive, sensorimotor and salience networks, may indicate a tendency toward excessively negative self-reflection and constitutes a significant disturbance in self-organization. Indeed, dysphoric and depressive symptoms are characterized by heightened self-referential mental activity, i.e., rumination, which amplifies disconnection from embodied and interoceptive present experience [106]. This recursive pattern reflects a shift in the balance of self-organization, in which mental self-processing predominates over bodily and sensory dimensions, to which individuals may be hypersensitive yet insufficiently affectively attuned, thereby narrowing engagement with both the body and the external world. Such network imbalance, manifesting as excessive overthinking alongside reduced embodied integration, has also been associated with obsessive-compulsive symptoms and eating disorders [107]. Psychoanalytically, excessive self-reflection may be understood as a defensive foreclosure of embodied and relational experience, in which thinking replaces transformation, echoing Bion’s distinction between thinking as beta-elements evacuation and thinking as alpha-function symbolization [72,108].

Working through disruptions of the self in psychotherapy involves the recognition and gradual reorganization of predictive, embodied, and relational processes. This work unfolds through the therapist’s attuned witnessing of subtle bodily affective features in the patient and within the therapeutic relationship. Such embodied relational engagement may allow boundaries to temporarily dissolve toward moments of experiential connection or merger, and subsequently re-emerge in the service of observation, mentalization, and differentiated relatedness. From an embodied predictive perspective, psychotherapy may be conceptualized as a temporally extended relational field in which heightened threat-based predictions can gradually become modulated through repeated experiences of co-regulation, affective attunement, and the greater rhythmic continuity across bodily and interpersonal experience. While direct evidence linking psychotherapy to changes in PPS organization or embodied temporal continuity remains limited, converging findings from interpersonal neuroscience and psychotherapy research suggest that repeated embodied relational experiences may support greater flexibility in self-related predictive and affective processes. However, longitudinal studies directly examining whether such embodied and relational mechanisms predict psychotherapeutic outcomes are still scarce.

From a psychodynamic perspective, within such a holding environment, intense interoceptive and sensorimotor states can be tolerated without immediate discharge or defensive evacuation, allowing them to undergo transformation into thinkable, symbolizable experience. Over time, this process may support a rebalancing between salience-driven immediacy and reflective integration, facilitating renewed coordination between bodily sensation, affective meaning, and autobiographical memory. In this sense, psychotherapy may facilitate self-modeling processes, potentially enabling corrective prediction errors and the generation of new anticipatory models, while progressively engaging and elaborating the self’s multiple dimensions within the therapeutic relationship.

## 6. Summary and Conclusions

Synthesizing neuroscience and psychodynamic perspectives, this paper has articulated selfhood as a multidimensional field of embodied relational organization. Across its sections, the self is conceptualized as a dynamically maintained configuration based upon interpersonal engagement and emerging from the continuous coordination of bodily states, action, affect, spatial orientation and temporal extension. PPS is conceptualized here as an embodied domain of self-organization, integrating somatosensory, sensorimotor, affective, social signals into a lived field of self-world boundaries within which subjectivity can arise, enabling the psyche to indwell the soma and anticipate action possibilities. Through salient and rewarding engagement, subjective time emerges as an embodied and relational process, shaped by entwined rhythms, interpersonal synchronization, and predictive engagement with affordances. Importantly, the present framework distinguishes between immediate sensorimotor anticipation within PPS, embodied interpersonal rhythms, and more extended autobiographical and narrative forms of temporal organization. In this way, spatial immediacy is progressively transformed into experiential continuity.

Building on this embodied temporal continuity, autobiographical and narrative dimensions of the self emerge as further elaborations of the self’s embodied organization across time. When embodied self-regulation and relational continuity become sufficiently stabilized, immediate experience may gradually extend into reflective space, enabling the consolidation of lived moments into long-term memory and their subsequent mental traversal. Autobiographical memory and mental time travel are framed as active inferential processes, supported by hippocampal–DMN integration, through which lived experience is organized into meaningful temporal sequences that guide present perception and future-oriented action. Reflective and narrative self-experience remains grounded in embodied regulation, interoceptive rhythms, and relational scaffolding, allowing temporal extension to unfold in continuity with bodily presence.

In alignment with neuroscientific accounts, psychodynamic perspectives further illuminate how symbolic capacity depends on a holding and containing environment, within which the dimensional expansion of experience enables the self to tolerate absence, anticipation, and symbolic transformation across time. Concepts such as transitional space, the capacity to be alone, dimensionality, and alpha function converge with neuroscientific findings to highlight that temporally extended selfhood depends on an integrated embodied base and the internalization of reliable relational presence. Developmental attachment research provides converging support for this view by demonstrating how early embodied caregiving experiences shape large-scale brain networks that support reflective self-experience and autobiographical organization across the lifespan.

Although the empirical specificity of these mechanisms varies across conditions, within this integrative framework psychopathology is understood as specific reorganizations and imbalances among self-dimensions. Neurodevelopmental conditions and traumatic states emphasize immediacy, bodily salience, and hyperactive readiness, whereas depressive rumination reflects a predominance of DMN-mediated self-reflection relative to embodied and relational engagement. These patterns underscore that disturbances of the self involve constrained PPS flexibility, defensive modes of self-evidencing, and altered coordination across self-dimensions.

Clinically, this model conceptualizes psychotherapy as a relational process that may support greater multidimensional integration and temporal mobility through sustained relational engagement. Therapeutic change unfolds within a temporally extended relational field in which embodied co-regulation, rhythmic continuity, and affective attunement allow connection to be established within the patient’s lived experiential organization. From this shared ground, relational embodied predictive processes can be gradually modulated, enabling bodily sensation and affective salience to become increasingly tolerable and available for reflective meaning-making. This process may further support imagination, autobiographical memory, and future-oriented agency. In this sense, therapeutic time may facilitate the progressive elaboration of a multidimensional self-model, allowing lived experience to be held in mind, remembered, and reworked across past, present, and anticipated future while sustaining its embodied grounding.

Importantly, the present framework integrates findings that differ in their degree of empirical support. While substantial evidence supports the embodied and relational dimensions of selfhood, including interoceptive processing, bodily self-consciousness, interpersonal synchrony, and autobiographical self-related network dynamics, several broader integrative proposals advanced here remain heuristic in nature. In particular, the proposed links between PPS organization, multidimensional self-processes, and psychotherapeutic transformation require further direct empirical investigation. Accordingly, the present framework is intended not as a definitive mechanistic account, but as an interdisciplinary and hypothesis-generating model designed to guide future process-oriented research at the interface of neuroscience, embodiment, and psychotherapy.

Future research should empirically examine the multidimensional framework proposed here by investigating neural dynamics related to changes in PPS flexibility, embodied temporal continuity, and relational attunement over the course of psychotherapy, and their clinical outcomes [53,109]. Multimodal process-oriented studies could integrate behavioral and physiological measures, like heart rate variability (HRV), autonomic rhythms and kinematic analyses of therapist–patient movement synchrony [110], along with neural data via mobile neuroimaging (EEG/fNIRS) or using holistic Mobile Brain-Body Imaging (MoBI) [111], to reveal dimensions of interactions driving change [112]. Mixed-methods designs, combining neurophysiological measures with qualitative interviews of therapists and patients, would enrich mechanistic insights and advance psychotherapy by pinpointing pathways of transformation [112].

In conclusion, the integration of neuroscience and psychodynamic perspectives deepens and grounds our understanding of the multidimensional self and the central role of embodied experience and relationship. By situating self-experience within lived bodily and interpersonal contexts, this dialogue illuminates both the organization and vulnerability of selfhood, and underscores the integrative potential of psychotherapy as a space in which brains–bodies–minds meet, resonate, and co-evolve across space and time.

## Data Availability

No data was used for the research described in the article.

## Abbreviations

The following abbreviations are used in this manuscript:

PPS	Peripersonal Space
DMN	Default Mode Network
mPFC	medial prefrontal cortex
PCC	posterior cingulate cortex
MoBI	Mobile Brain–Body Imaging
HRV	Heart Rate Variability
fNIRS	Functional Near-Infrared Spectroscopy
EEG	Electroencephalography
